# Simultaneous Super-Resolution and Classification of Lung Disease Scans

**DOI:** 10.3390/diagnostics13071319

**Published:** 2023-04-02

**Authors:** Heba M. Emara, Mohamed R. Shoaib, Walid El-Shafai, Mohamed Elwekeil, Ezz El-Din Hemdan, Mostafa M. Fouda, Taha E. Taha, Adel S. El-Fishawy, El-Sayed M. El-Rabaie, Fathi E. Abd El-Samie

**Affiliations:** 1 Department of Electronics and Communications Engineering, High Institute of Electronic Engineering, Ministry of Higher Education, Bilbis-Sharqiya 44621, Egypt; 2 School of Computer Science and Engineering (SCSE), Nanyang Technological University (NTU), Singapore 639798, Singapore; 3 Security Engineering Lab, Computer Science Department, Prince Sultan University, Riyadh 11586, Saudi Arabia; 4 Department of Electronics and Electrical Communications Engineering, Faculty of Electronic Engineering, Menoufia University, Menouf 32952, Egypt; 5 Department of Computer Science and Engineering, Faculty of Electronic Engineering, Menoufia University, Menouf 32952, Egypt; 6 Department of Electrical and Computer Engineering, Idaho State University, Pocatello, ID 83209, USA; 7 Department of Information Technology, College of Computer and Information Sciences, Princess Nourah Bint Abdulrahman University, Riyadh 11564, Saudi Arabia

**Keywords:** Coronavirus, chest X-ray radiographs, convolutional neural network, image super-resolution, multi-class SVM

## Abstract

Acute lower respiratory infection is a leading cause of death in developing countries. Hence, progress has been made for early detection and treatment. There is still a need for improved diagnostic and therapeutic strategies, particularly in resource-limited settings. Chest X-ray and computed tomography (CT) have the potential to serve as effective screening tools for lower respiratory infections, but the use of artificial intelligence (AI) in these areas is limited. To address this gap, we present a computer-aided diagnostic system for chest X-ray and CT images of several common pulmonary diseases, including COVID-19, viral pneumonia, bacterial pneumonia, tuberculosis, lung opacity, and various types of carcinoma. The proposed system depends on super-resolution (SR) techniques to enhance image details. Deep learning (DL) techniques are used for both SR reconstruction and classification, with the InceptionResNetv2 model used as a feature extractor in conjunction with a multi-class support vector machine (MCSVM) classifier. In this paper, we compare the proposed model performance to those of other classification models, such as Resnet101 and Inceptionv3, and evaluate the effectiveness of using both softmax and MCSVM classifiers. The proposed system was tested on three publicly available datasets of CT and X-ray images and it achieved a classification accuracy of 98.028% using a combination of SR and InceptionResNetv2. Overall, our system has the potential to serve as a valuable screening tool for lower respiratory disorders and assist clinicians in interpreting chest X-ray and CT images. In resource-limited settings, it can also provide a valuable diagnostic support.

## 1. Introduction

Lower respiratory diseases are a significant cause of mortality in developing countries, with acute lower respiratory infections being the main cause of death. Despite the development of various diagnostic and therapeutic strategies, the lack of access to high-quality health care in resource-limited settings has resulted in delayed diagnosis and treatment of diseases. This delay can result in the spread of infectious diseases, the development of complications, and ultimately, increased morbidity and mortality. In addition, the COVID-19 pandemic has further highlighted the need for rapid and accurate diagnostic tools for respiratory diseases. The pandemic has overwhelmed healthcare systems globally, and the lack of effective screening and diagnostic tools has made it difficult to control the spread of the disease. Lung diseases are disorders of the lung airways and other structures [1]. Pneumonia, tuberculosis (TB), and corona-virus disease 2019 (COVID-19) are examples of lung ailments. Lung diseases are the cause of mortality for millions of individuals, according to the Forum of International Respiratory Societies (IRS) [2]. Every year, 1.4 million people die from TB, and millions more die from pneumonia. Furthermore, COVID-19 affects the whole world [3], where it has infected millions of individuals, overwhelming healthcare systems in several nations [4].

The development of a computer-aided diagnostic system for common lower respiratory diseases, using chest X-ray and CT images, is therefore significant as it can provide an accessible and affordable screening tool for resource-limited settings [5,6]. It can help healthcare providers make faster and more accurate diagnoses, and ultimately improve diagnosis outcomes. Furthermore, the use of DL techniques and SR reconstruction can enhance the accuracy of the system and potentially reduce the need for additional diagnostic tests. The CT is a medical imaging technique that uses computer analysis to generate high-resolution sub-images of a person’s body from different angles. These sub-images can be viewed individually or combined to create a three-dimensional representation of the patient’s organs, tissues, bones, and any abnormalities. In comparison, X-ray scans are less detailed than CT scans. The CT scans are utilized in various studies to identify lung diseases, such as pneumonia, lung cancer, and COVID-19 [7,8,9,10].

Lung diseases have traditionally been diagnosed through skin tests, blood tests, and sputum sample tests [11], as well as chest X-ray tests and CT scan tests [12]. Since COVID-19 primarily affects the lungs, medical imaging techniques such as X-ray and CT scans are frequently utilized to assess the severity of infection [13,14]. X-ray imaging is extensively employed in the diagnosis of various lung disorders due to availability, low processing time, and low cost. However, CT imaging is recommended, because it provides more information about the affected areas [15].

Chest X-ray imaging is a popular modality for lung assessment [16]. It helps to identify lung cancer, infections, and pneumothorax, a condition that occurs due to the accumulation of air around the lungs causing them to collapse [17]. In addition, X-ray imaging is a cost-effective and simple technology that emits lower radiation than CT scans [18]. Although X-ray imaging carries a risk of radiation exposure, it is highly beneficial for diagnosis, when employed in the safest and most regulated settings. However, the images produced are of average quality, and 3D information is not available. To enhance image quality, pre-processing techniques should be implemented.

To minimize the spread of COVID-19 and ensure prompt treatment, early identification of the virus is essential, and differentiation from other lung disorders is crucial. Currently, the most effective measure to prevent COVID-19 transmission is to isolate and quarantine suspected cases. A study in [19] demonstrated the feasibility of using computer techniques and CT scans to differentiate COVID-19 from pulmonary edema. EDECOVID-net was developed. It automatically distinguishes between COVID-19 infections and those induced by edema in CT images. The findings revealed that EDECOVID-net achieves an accuracy of 98% in distinguishing patients with COVID-19 from those with pulmonary edema.

Lung cancer is a highly-fatal cancer, but its early detection can significantly improve treatment outcomes. However, the unpredictability of lung cancer nodules poses a challenge for computer-aided automated activities. Faruqui et al. proposed a hybrid deep convolutional neural network-based model called LungNet. It was trained on both CT scans and wearable sensor-based medical IoT (MIoT) data. LungNet is a 22-layer CNN that combines latent properties of CT scan scans and MIoT data to increase diagnostic accuracy. The network, which is managed by a centralized server, was trained on a balanced dataset of 525,000 images, and it achieved an accuracy of 96.81% in classifying lung cancer into five categories.

Machine learning (ML) and DL have great potential in the diagnosis of various disorders, including lung diseases, by analyzing medical imagery. Recent advances in ML and DL, particularly in the classification of time series and medical images, have shown promising results [20,21,22,23]. The DL approaches can extract features directly from raw data, which aids in accurately detecting some ailments [24]. Deep convolutional neural networks (DCNNs) are considered state-of-the-art classifiers to be used in medical applications. Support vector machines (SVMs) are well-known for their effectiveness in classification and regression [10,25,26]. Shuhua et al. [27] developed a technique for assessing the error in kernel regularized regression using a non-convex loss function, which minimizes the negative impact of outliers on its performance. Despite the experience of radiologists, predicting infections using medical imaging is challenging due to the lack of detailed disease knowledge. Combining medical images with DL algorithms is a viable option in detecting lung diseases [28,29].

Deep learning (DL) is a popular approach used in various fields, including computer vision, natural language processing, and speech recognition. In the context of X-ray and CT image SR and classification for lung diseases, DL can provide significant benefits. For instance, DL-based SR techniques can improve the resolution and details of X-ray and CT images, enabling more accurate diagnosis and detection of lung diseases. Additionally, DL models can automatically learn and extract relevant features from X-ray and CT images, without the need for manual feature engineering. This can enhance the accuracy of lung disease classification. Furthermore, DL models are capable of processing and analyzing large and complex datasets, which are common in medical imaging. As a result, DL-based classification models can analyze X-ray and CT images and provide accurate diagnosis and classification of lung diseases in a fraction of the time compared to manual diagnosis by radiologists. This can lead to earlier detection and treatment, which improves diagnosis outcomes and reduces healthcare costs. Finally, DL-based models can analyze large amounts of patient data to develop personalized treatment plans. This paper presents a computer-aided diagnostic system from chest X-ray and CT images for several common pulmonary diseases, including COVID-19, viral pneumonia, bacterial pneumonia, TB, lung opacity, and various types of carcinoma. The proposed system depends on SR techniques to enhance image details and DL techniques for both SR reconstruction and classification. The InceptionResNetv2 model was used as a feature extractor in conjunction with an MCSVM classifier. The paper gives a comparison of the proposed model performance with those of other classification models, such as Resnet101 and Inceptionv3, and gives an evaluation of the effectiveness of using both softmax and MCSVM classifiers. The proposed system was tested on three publicly-available datasets of CT and X-ray images.

The main contributions of this paper are summarized as follows:

- Presenting a DL framework for diagnosis of lung diseases from chest X-ray and CT images.
- Studying the impact of image SR on lung disease diagnosis.
- Presentation of InceptionResNetv2 as a feature extractor and comparing its results with those of Resnet101 and Inceptionv3 models.
- Investigation of the proposed framework in five-class and six-class scenarios using softmax and MCSVM classifiers.

The structure of this paper consists of several sections. Section 2 provides an overview of the related work, highlighting the current state-of-the-art techniques in the field of computer-aided diagnosis of lung diseases. Section 3 introduces the proposed classification framework, outlining the SR and DL techniques utilized for classification. In Section 4, experimental results are presented, providing an evaluation of the proposed system performance on three publicly available datasets of CT and X-ray images. Section 5 offers a discussion and comparison of the proposed framework with other classification models. Finally, Section 6 presents the final remarks, summarizing the key findings, contributions, and potential applications of the proposed system.

## 2. Related Work

The DL provides considerable promising solutions for detecting various disorders [30,31,32,33,34]. Xu et al. [35] presented a DL-based system for analyzing COVID-19 images. Using the DL model, the possible infection sites are separated into logged trees. On CT scans of COVID-19, viral pneumonia, and normal patients, a three-class classification problem was established. Using the Bayesian algorithm, the infection type and overall confidence score were obtained. This system achieved an accuracy of 86.7%. Using radiomic texture descriptors, Chandra et al. [36] proposed an automated technique for COVID-19 identification. Their technique was tested on X-ray images. The authors reported an accuracy of 91.329%.

Alqudah et al. [37] developed a hybrid AI system that can detect COVID-19 from chest X-ray images by combining different AI techniques, including DL (CNN with softmax classifier) and ML (SVM, KNN, and RF) classifiers [38]. The results showed that the presented methodology is efficient and useful in detecting COVID-19 in just a few seconds. The obtained results proved that the performance of all classifiers is good and most of them recorded accuracy, sensitivity, specificity, and precision of more than 98%. Bhowmik et al. [39] presented a multi-modal approach for real-time COPD exacerbation prediction. It includes a spatio-temporal AI architecture for cough detection using data from sensor networks, and exacerbation prediction. In addition to demonstrating the viability of implementing a passive, continuous, remote patient monitoring and telehealth solution for chronic respiratory diseases, the researchers developed an early warning system based on AI and multi-factor analysis to decrease hospitalizations and medical costs.

To increase the effectiveness and accuracy of diagnosis, the EfficientNetv2-M model was designed and used to diagnose lung disorders on X-ray images using pre-trained weights from ImageNet [40]. The dataset was first augmented. The augmentation results were then automatically fed into a DL model to extract their important features for classifying diseases. This model produced validation results of 82.15% for accuracy and predicted the three classes of normal, pneumonia, and pneumothorax in the NIH dataset. Additionally, the obtained results for the four classes of the SCH dataset, namely normal, pneumonia, pneumothorax, and TB revealed a validation accuracy of 82.20%. To analyze CT scans and X-ray images, the researchers in [41] used pre-implemented instances of a CNN and Darknet. CNN and Darknet with image processing algorithms enable the analysis, identification, and localization of anomalies in CT scans and X-ray images. They produced a 98% accuracy with a loss value of just 0.04.

Rasheed et al. [42] studied how to use feature selection methods and transfer-learning (TL) networks to increase the classification accuracy of ML classifiers. To produce significant features from images, three different TL networks—AlexNet, ResNet101, and SqueezeNet—were evaluated. By using feature-selection techniques such as iterative neighborhood component analysis (iNCA), iterative chi-square (iChi2), and iterative maximum relevance-minimum redundancy (iMRMR), the obtained relevant features were further refined. The classification process was completed by employing SVM, CNN, and linear discriminant analysis (LDA) classifiers. The combination of AlexNet, ResNet101, SqueezeNet, iChi2, and SVM produced a classification accuracy of 99.2%, when used to classify X-ray images. Similarly, a 99.0% accuracy was produced by AlexNet, ResNet101, SqueezeNet, iChi2, and the presented CNN network. Hong et al. [43] suggested a CNN-based multi-class classification technique for lung diseases. A classification model of the multi-GAP format was constructed based on the noisy student ImageNet pre-trained weights of the EfficientNet B7 model. On the dataset of Soonchunhyang University Hospital in Cheonan, an average accuracy of 96% was achieved. To reduce the mean square error, Pradhan et al. [44] proposed a lung cancer diagnostic model. Principal component analysis (PCA) and t-distributed stochastic neighbor embedding (t-SNE) have been used for feature extraction. Additionally, a self-adaptive sea lion optimization algorithm (SA-SLnO) that employs the most recent meta-heuristic algorithms to optimize the weights has been presented as an improved correlation-based weighted feature extraction algorithm. The presented SA-SLnO maximizes the number of hidden neurons in RNN. In addition, researchers in [45] developed a method for identifying lung cancer. Two widely-used methods, namely PCA and t-SNE, have been applied to extract features. Furthermore, deep features have been obtained from the CNN pooling layer. Additionally, the best fitness-based squirrel search algorithm (BF-SSA) has been used to determine the most important features. This hybrid optimization method is regarded as being superior in many fields for effectively exploring the search space and improving the feature selection performance. High ranking deep ensemble learning (HR-DEL) is used for five types of detection models in the final step. In addition, the final anticipated output is produced based on the high ranking of all classifiers.

Souid et al. [46] proposed a modified model, namely MobileNetV2, to classify and predict lung diseases in frontal thoracic X-ray images. A combination between TL and metadata leveraging has been presented. The presented model was tested on the NIH Chest-Xray-14 database, and it provided a 90% accuracy. The TL models were used to develop a COVID-19 prediction model for chest CT scans in [47]. Three common DL models, namely, VGG-16, ResNet50, and Xception were utilized. Then, a method for combining the aforementioned pre-trained models was described in order to increase the system total capacity for prediction. The presented model has a classification accuracy of 98.79%.

For detecting COVID-19 from chest X-ray images, an automated DL classification approach was presented in [48]. Prior to applying CNN models on the dataset, histogram equalization, spectrum, greys, and cyan were used to improve the performance. The COVID-19 symptoms were recently identified using the available dataset and eleven pre-existing CNN models: VGG16, VGG19, MobileNetV2, InceptionV3, NFNet, ResNet50, ResNet101, DenseNet, EfficientNetB7, AlexNet, and GoogLeNet. Among all deployed CNN models. It was discovered that the modified MobileNetV2 model provides the highest accuracy of 98% in classifying COVID-19 and healthy chest X-ray images.

Rahman et al. [49] developed a framework for detecting bacterial and viral pneumonia in X-ray images. For the classification procedure, various pre-trained models, such as AlexNet, ResNet18, DenseNet201, and SqueezeNet, were employed. This framework yielded COVID-19-normal and COVID-19-pneumonia classification accuracy levels of 98% and 95%, respectively. Furthermore, for three-class classification, an accuracy of 93.33% was attained.

Ferreira et al. [50] developed a system for classifying pneumonia from chest X-ray images. A binary mask was created using a pre-trained U-Net-based TL model. For the classification, VGG-16 was employed. Rania et al. [51] demonstrated a DL model for detecting COVID-19 in X-ray images. Their concept is built upon a single-shot detector (SSD) and a residual network (ResNet101). Firstly, X-ray images were pre-processed and augmented. After that, ResNet101 was used for classification, and it achieved an accuracy of 94.95%.

Zhang et al. [52] used a combination of CT and X-ray scans to better diagnose COVID-19. Using the convolutional block attention module, a deep convolutional attention network (MIDCAN) with multiple inputs was created. The first input receives 3-D CT images, while the second receives 2-D X-ray images. The sensitivity of their presented system was 98.10%, the specificity was 97.95%, and the accuracy was 98.02%. Wang et al. [53] introduced an AI method for COVID-19 classification from CT images. Pre-trained models were used to learn features, and a transfer feature learning approach was utilized to extract features. A pre-trained network selection strategy for fusion was presented in order to determine the best two models. Discriminant correlation analysis was used to aid in the feature fusion of the two models’ features using deep CT fusion. COVID-19-pneumonia, COVID-19-TB, COVID-19-normal, and pneumonia-normal classification states were implemented with accuracy levels of 97.32%, 96.42%, 96.99%, and 97.38%, respectively.

Different CNN-based models have lately shown promising performance levels in the challenge of single image super-resolution (SISR). On the other hand, several cutting-edge SISR approaches employ tactics that are effective in other vision tasks. He et al. [54] employed a 22-layer multi-receptive-field network (MRFN) to completely learn the LR-to-HR mapping function. The multi-receptive-field module serves as a foundation for learning of object mappings. It takes different properties from small, middle, and large receptive fields and combines them into a module. Furthermore, instead of using the L1 and L2 loss functions, the weighted Huber loss, a two-parameter training loss, is utilized to adaptively adjust the value of the back-propagated derivative according to the residual value.

Mehrrotraa et al. [55] presented a DL-based method to identify TB. This presented method involves ensemble efficient deep convolutional networks and ML algorithms, which do not require heavy computational costs. The model achieved accuracy levels of 87.90% and 99.10% with an AUC values of 0.94 and 1, respectively, in identifying TB-infected images from normal and COVID-infected images. The authors of [56] proposed a completely automated framework with a DL model for the recognition and classification of chronic pulmonary disorders and COVID-pneumonia using chest X-ray images. This framework consists of a three-step process that extracts the region of interest, detects infected lungs, and classifies the images into COVID-pneumonia, pneumonia, and other chronic pulmonary disorders. This framework achieved an accuracy of 96.8% in classifying lung images.

Masad et al. [57] presented a hybrid DL system comprising a CNN model with additional classifiers (SVM, k-nearest neighbor (KNN), and random forest (RF)) for automated pneumonia detection. Although the hybrid systems demonstrate comparable performance to that of the traditional CNN model with softmax layer in terms of accuracy, precision, and specificity, the RF hybrid system performed less efficiently than the others. Although the KNN hybrid system showed the best consumption time, sensitivity was sacrificed to achieve this target. However, this new hybrid methodology achieved high efficiency and a short classification time for detecting pneumonia from small-size chest X-ray images. Limitations of this study include the use of only small-size chest X-ray images and potential challenges in scaling the approach to larger image datasets.

Al-Issa et al. [58], discussed the difficulties of accurately diagnosing various pulmonary diseases, which have similar radiographic characteristics. To address this target, the authors explored the performance of four popular pre-trained models (VGG16, DenseNet201, DarkNet19, and XceptionNet) in distinguishing between normal, pneumonia, COVID-19, and lung opacity cases from chest-X-ray images. The XceptionNet model outperformed all other ones, achieving a 94.775% accuracy and an AUC of 99.84%. DarkNet19 provided a good compromise between accuracy, fast convergence, and resource utilization. Ensemble features allowed to achieve the highest accuracy of 97.79% among all surveyed methods, but it took the largest time to predict an image (5.68 s). The authors suggested that an efficient and effective decision support system could be developed using these approaches to assist radiologists in accurately assessing pulmonary diseases in various healthcare sectors. The study also focused solely on chest radiographs and did not cover the potential benefits of using other imaging modalities, such as CT scans. Finally, the study is limited in that it is only concerned with performance evaluation of the models on a specific set of pulmonary diseases. The models were not applied on other diseases or conditions.

## 3. Materials and Methods

Various datasets are used to assess the proposed DL framework. The description of these datasets is presented in Table 1. The dataset #1 is a collection of COVID-19, normal, pneumonia-viral, pneumonia-bacterial, and TB chest X-ray images gathered from open-source Kaggle datasets. The dataset #1 [59] has 259 X-ray images of COVID-19 patients and 1000 X-ray images of healthy people. The dataset #2 [59] has 900 X-ray images of pneumonia patients with bacterial pneumonia and 800 X-ray images of pneumonia patients with viral pneumonia. The dataset #3 [60] has 800 X-ray images of TB patients. The DL models often require a huge amount of data to be trained. The more data the network encounters during training, the better it can learn to distinguish different disease representations. Hence, image augmentation strategies are exploited to obtain a large amount of data for the training process. For the COVID-19 dataset, different augmentation strategies are used. The dataset is increased to 1000 X-ray images for each class after augmentation.

In addition to the dataset #1, We selected data from six different available datasets [61,62,63,64,65,66] to create a big lung X-ray and CT scan dataset for lung disease detection. These datasets have been utilized publicly for lung disease diagnosis and have demonstrated appropriateness for DL applications. As a result, by learning from all of these resources together, the combined dataset is expected to improve the generalization ability of the proposed DL model. The X-ray scan dataset #2 consists of 35,399 images belonging to 5 different classes that have been used to evaluate the proposed framework. The dataset contains 3616 X-ray scans for COVID-19 cases, 6012 X-ray scans for lung opacity, 10,192 X-ray scans for normal cases, 8624 X-ray scans for TB cases, and 3080 X-ray scans for viral pneumonia cases. In addition, the CT scan dataset #3 consists of 28,058 images belonging to the 6 different classes that have been used to evaluate the proposed framework. The dataset contains 7942 CT scans for COVID-19 cases, 7290 CT scans for non-COVID-19 cases, and three different chest cancer types (4290 CT scans for adenocarcinoma, 2508 CT scans for large cell carcinoma, and 3410 CT scans for squamous cell carcinoma) and 2618 images for community-acquired pneumonia (CAP) cases.

### 3.1. The Proposed Framework

**Figure 1:**
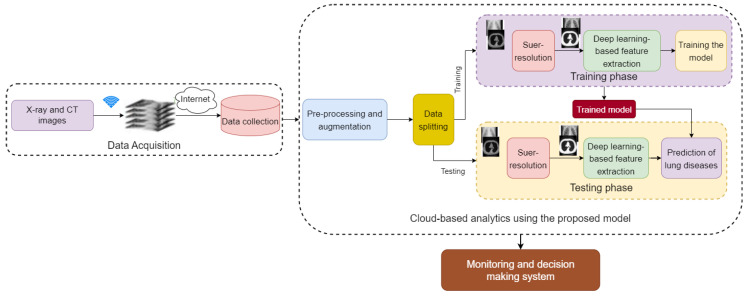
Main architecture of the proposed framework.

The proposed framework aims to make it possible for those suffering from lung disorders to live, securely. Furthermore, it offers efficient supporting settings managed by caregivers, such as friends, family, and medical staff. This may be accomplished by leveraging contemporary technologies such as cloud computing, and AI to monitor people infected with lung diseases in real time in streets or workplaces. As a result, this system delivers dependable and timely healthcare services for patient monitoring. The main objective of the suggested framework is to keep track of lung conditions. Patients’ data are acquired via wearable and portable devices. Then, data records are produced on the cloud, and finally, authorized healthcare workers get access to this data at any time and from any location. This architecture may aid in the provision of remote lung disease monitoring. The proposed framework is divided into three stages that work together to achieve the system target. Every stage performs a certain function that works in tandem with the others. Figure 1 shows the proposed framework with three stages. Data acquisition, cloud-based analysis using the proposed model, monitoring and decision-making are the stages of the proposed framework.

### 3.2. Data Acquisition

In this phase, data acquisition devices work in real time. X-ray and CT images are obtained from different online accessible resources. A wireless network of specialized image acquisition systems can be utilized to gather images inside a smart hospital system. Afterwards, the gathered images are forwarded to a gateway. This gateway is used between the wireless network and the server hosted in the cloud for healthcare disease prediction in a decision-making step. The controller sends the gathered images to the respective channel periodically via a communication protocol such as MQTT.

### 3.3. Cloud-Based Analysis Using the Proposed Models

**Figure 2:**
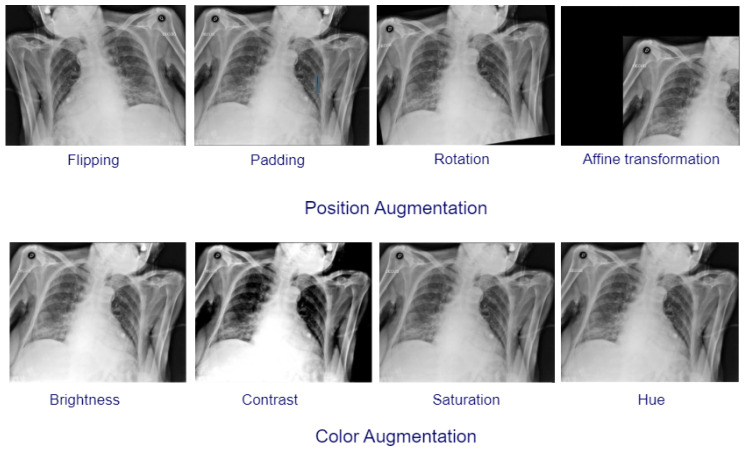
Samples of augmented images.

When patient lung disease data are received via the Internet and sorted, they are then made available for review by professionals using a community of processing and storage capabilities provided by the cloud. Image pre-processing and augmentation are important steps in preparing X-ray and CT images for lung disease classification. We have resized the X-ray and CT image dimensions to 299×299×3 in order to match the required size of input to the three proposed models. For dataset #1 the COVID-19 images are augmented to obtain 1000 images from 256 images. The augmentation operations include position augmentation and color augmentation. Figure 2 presents samples for augmented images. In position augmentation, the pixel positions of an image are changed, while color augmentation deals with the color properties of an image by changing its pixel values.

#### 3.3.1. Image Super-Resolution

**Figure 3:**
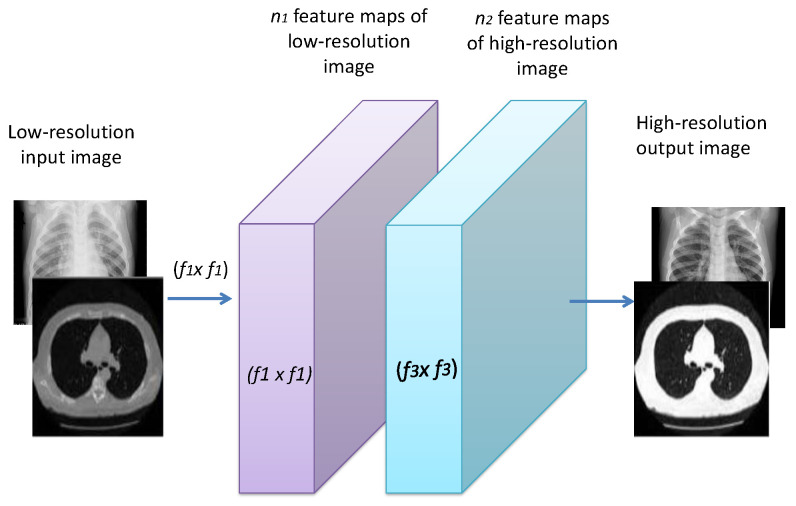
Block diagram of the proposed DCNN image SR model.

Image SR is used mainly to produce an HR image from an LR one through a mapping process. In this paper, the mapping is implemented by DCNN. The main aim is to recover an F(Y) image from the LR image *Y*, where F(Y) and the ground-truth HR image *X* should be as identical as possible. Figure 3 presents a lightweight CNN model for image SR. The mapping *F* mainly consists of three processes:

- **Patch extraction and representation:** Patches from the LR image *Y* are extracted, and then each patch is represented as a high-dimensional vector. This can be expressed as:
  (1) $$F_{1}\left(Y\right)=max\left(0,W_{1}\times Y+B_{1}\right)$$
  where $W_{1}$ represents the weights for the first convolution layer, which has a size of $c\times f_{1}\times f_{1}\times n_{1}$. *c*, $f_{1}$, and $n_{1}$ are the numbers of channels for the input image, the spatial filter size, and the number of filers, respectively. A rectified linear unit (ReLU) is applied on the output to add non-linearity.
- **Non-linear mapping:** An $n_{1}$-dimensional feature vector is extracted for each patch from the first layer. Then, these $n_{1}$-dimensional feature vectors are mapped as $n_{2}$-dimensional vectors. This mapping can be represented as:
  (2) $$F_{2}\left(Y\right)=max\left(0,W_{2}\times Y+B_{2}\right)$$
  where $W_{2}$ has a size of $n_{1}\times 1\times 1\times n_{2}$. Each of the output $n_{2}$-dimensional vectors are used for reconstruction.
- **Reconstruction:** A pre-defined filter that acts as an averaging filter for the reconstruction process is used. The last convolutional layer is exploited to obtain the final HR image. The reconstruction process can be expressed as:
  (3) $$F\left(Y\right)=W_{3}\times F_{2}\left(Y\right)+B_{3}$$
  Mean squared error (MSE) is used as the loss function L(Θ).
  (4) $$L\left(\Theta \right)=\frac{1}{k}\sum_{i=1}^{k}{∥F\left(Y_{i};\Theta \right)-X_{i}∥}^{2}$$
  where *k* represents the number of training samples.

In this paper, the filter sizes are chosen to be $f_{1}=9$ and $f_{2}=3$ with numbers of filters $n_{1}=32$ and $n_{2}=16$. A Gaussian distribution with μ=0 and σ=0.001 is used to initiate the weights randomly, with 0 bias and $10^{-5}$ learning rate as in [67].

#### 3.3.2. DL-Based Feature Extraction

**Figure 4:**
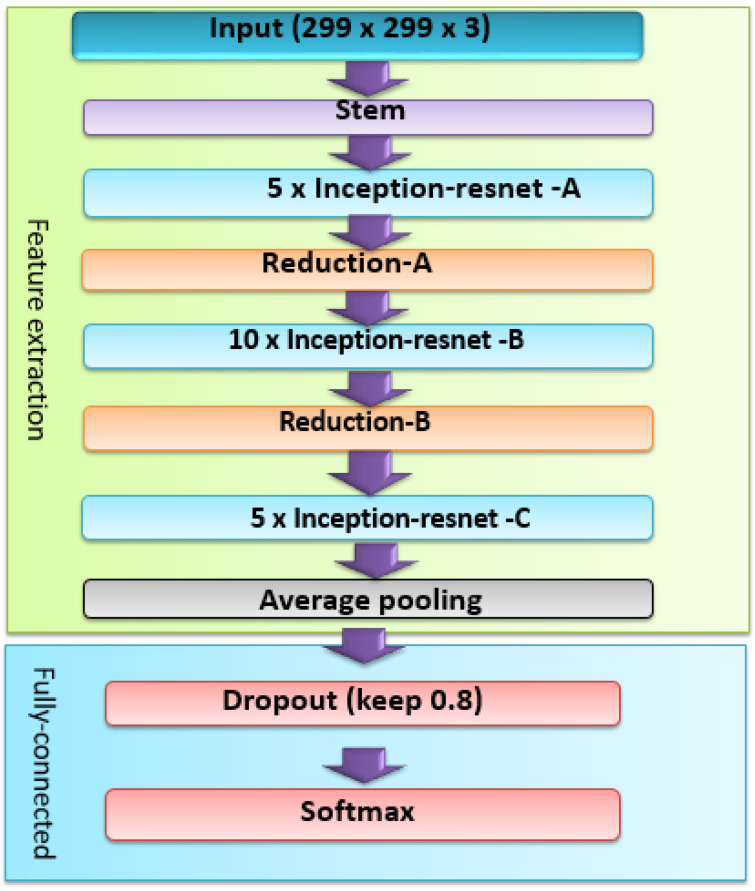
Overall scheme of the InceptionResNetv2 network.

DL-based feature extraction is a method of using deep neural networks to automatically learn and extract useful features from images or other data. DL-based feature extraction is widely used with medical images, such as X-ray and CT images. It is used in lung disease classification, as it is able to learn features that are specific to lung patterns and anomalies that represent certain diseases. In this paper, InceptionResNetv2 is used for feature extraction. It is a convolutional neural network that uses Inception architectures with residual connections. The residual connection replaces the filter concatenation stage. It has a 164-layer depth, an 299×299 input image size, and 1000 classes for the output [68]. However, in this paper, the fully connected layer is removed and the average pooling layer is employed as the final layer. The architecture of the whole system of InceptionResNetv2 is shown in Figure 4.

It is clear that the InceptionResNetv2 contains two sections, feature extraction, and a fully-connected layer.

**Stem module:** It represents the first layer of the architecture. It mainly consists of convolution (Conv) and max-pooling layers. The convolution filter in the Stem module is 3×3 in size and the stride value is 2. Therefore, the parameter values will be decreased, where the (299×299×3) input image size is converted into (35×35×384) [69].

**Figure 5:**
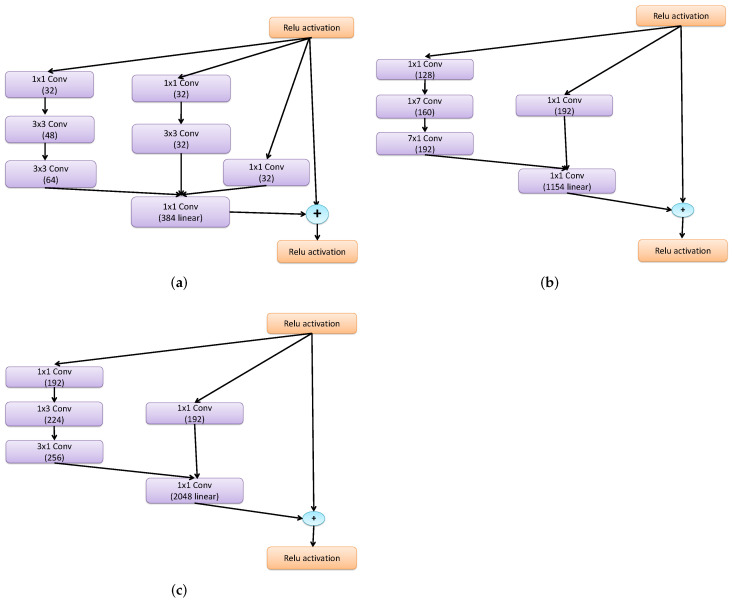
Overall architecture of the Inception-resnet modules. (**a**) Inception-resnet-A. (**b**) Inception-resnet-B. (**c**) Inception-resnet-C.

**Inception-resnet Modules:** The combination of the inception layer and residual connection is the advantage of the inception-resnet module. It contains three primary modules, denoted by the letters A, B, and C in Figure 5. Inception-resnets of the same kind are linked sequentially. A reduction module is required to link the inception-resnet with different types.

**Figure 6:**
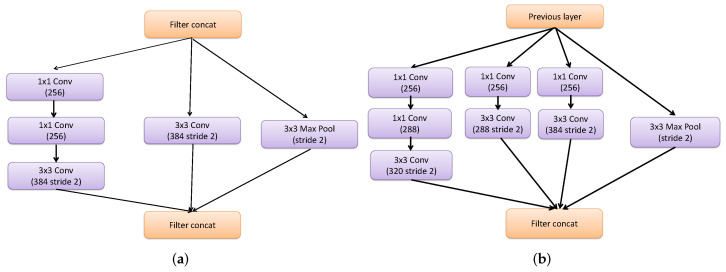
Reduction-A and Reduction-B modules (**a**,**b**).

**Reduction Modules:** The reduction module is used to reduce parameters between inception and resnet modules. As illustrated in Figure 6, the inception-resnet design contains two reduction modules, A and B. By transforming a 35×35 shape to a 17×17 shape, the Reduction-A module unites Inception-A and Inception-B modules. Furthermore, by reducing a 17×17 form to an 8×8 shape, the Reduction-B module links Inception-resnet-B and Inception-resnet-C modules. Finally, the average pooling layer converts the output of the Inception-resnet-C module into a 1-D vector of 1792 features. The InceptionResNetv2 is utilized as a feature extractor in this study, with an MCSVM classifier replacing the fully-connected layer.

#### 3.3.3. Proposed Classification Frameworks

(1) Softmax is the final layer at the network end. It generates the actual probability scores for each class label. In this paper, five-class and six-class classification problems are introduced. The softmax layer has *n* nodes marked as $p_{i}$, where i=1:n. $p_{i}$ represents the discrete probability distributions. The input to the softmax layer can be represented as follows:
  (5) $$a_{i}=\sum_{k}^{}h_{k}W_{ki}$$
  Then, $p_{i}$ can be calculated as:
  (6) $$p_{i}=\frac{exp(a_{i})}{\sum_{j}^{5}exp\left(a_{j}\right)}$$
  Then, the predicted class $\hat{i}$ can be obtained as follows:
  (7) $$\hat{i}=arg\;max\;p_{i}$$
  where *h* and *W* represent the activation and the weight of the layer nodes that precede the softmax layer, respectively.
(2) Multi-class Support Vector Machine Classifier: The SVM is a commonly used classifier for binary classification problems. It constructs decision hyperplanes that best divide the dataset into classes. For multi-class classification problems, the number of classes *M* is greater than two. The SVM uses several strategies to solve multi-class classification problems such as binary tree (BT), one-against-one (OAO), directed acyclic graph (DAG), and one-against-all (OAA) classifiers [70]. In this work, the OAASVM classifier with polynomial kernels is used as in [71]. *M* SVM models have been constructed, one for each class. The *m*th classifier is trained with all samples for class *m* and marked with positive labels, whereas the $M_{1}$ remaining classes are marked with negative labels. This gives advantages in terms of the short training time. The training of a single sub-classifier becomes much simpler.
  For *n* training data $\left(x_{1};y_{1}\right);:::;\left(x_{n};y_{n}\right),$ where $x_{i}\in R^{n};i=1;:::;n$ and $y_{i}\in 1;:::;M$ is the class of $x_{i}$. The class *m* SVM solves the following [72]:
  (8) $$min_{\omega^{m},b^{m},\zeta^{m}}\frac{1}{2}{\left(\omega^{m}\right)}^{T}\omega^{m}+C\sum_{1}^{n}\zeta_{i}^{m}$$
  $$\begin{array}{c} {\left(\omega^{m}\right)}^{T}\phi \left(x_{i}\right)+b^{m}\geq 1-\zeta_{i}^{m}\;if\;y_{i}=m\\ {\left(\omega^{m}\right)}^{T}\phi \left(x_{i}\right)+b^{m}\geq -1+\zeta_{i}^{m},\;if\;y_{i}\neq m\\ \zeta_{i}^{m}\geq 0,i=1,.....,n \end{array}$$
  where ϕ(x) is the mapping function. For nonlinear separation, a penalty term $C\sum_{1}^{n}\zeta_{i}^{m}$ is added for error reduction, where *C* represents the penalty parameter. In order to minimize the term $\frac{1}{2}{\left(\omega^{m}\right)}^{T}\omega^{m}$, the margin between two groups of data $2/∥\omega_{m}∥$ should be maximized. After solving Equation (8), there are *M* decision functions ${\left(\omega^{1}\right)}^{T}\phi \left(x\right)+b^{1}$, ............, ${\left(\omega^{M}\right)}^{T}\phi \left(x\right)+b^{M}$.
  (9) $$f\left(x\right)=argmax_{m=1,..,M}\left({\left(\omega^{m}\right)}^{T}\phi \left(x\right)+b^{m}\right)$$
  where f(x) is the decision boundary function. We can say that *x* belongs to a specific class that has the largest decision function value. The parameters of the SVM are presented in Table 2.

## 4. Experimental Results

In order to concentrate on the improvements obtained by image SR, this paper is concerned with the influence of image SR on three DCNN models used as feature extractors with an MCSVM classifier. We try to obtain a better performance model for the lung disease classification problem.

### 4.1. Evaluation Metrics

The confusion matrix is a table used to characterize the classifier performance. For multi-class classification, the one-against-all approach can be used to evaluate the classifier performance. In this paper, five classes are considered, COVID-19 (A), pneumonia-viral (B), pneumonia-bacterial (C), TB (D), and normal (E). In a 2×2, once class A is elected as positive, the other is automatically negative. In the case of the five-class classification, there are five different metrics depending on which of the five classes is elected as positive. The metrics can be calculated as in the 2×2 case, such as class A against not-Class A, then class B against not-class B, and so on. The overall performance is evaluated based on the sensitivity (Sen), specificity (Spec), accuracy (Acc), precision (Preci), Matthews correlation coefficient (Mcc), false positive rate ($F_{pr}$), and $F_{1}$ score [73]. The $T_{p}$ of A is all A cases that are classified as A, $T_{n}$ of A is all non-A cases that are not classified as A, $F_{p}$ of A is all non-A cases that are classified as A and $F_{n}$ of A is all A cases that are not classified as A. In order to find these four outcomes of B, C, D, and E classes, A is replaced with B, C, D, or E [74].

Sensitivity is given by:

(10) $$Sen=\frac{T_{p}}{T_{p}+F_{n}}\times 100$$

Specificity is given by:

(11) $$Spec=\frac{T_{n}}{T_{n}+F_{p}}\times 100$$

Accuracy is given by:

(12) $$Acc=\frac{T_{p}+T_{n}}{T_{p}+T_{n}+F_{p}+F_{n}}\times 100$$

Precision is given as:

(13) $$Preci=\frac{T_{p}}{T_{p}+F_{p}}$$

Matthews correlation coefficient (Mcc) is defined as:

(14) $$Mcc=\frac{T_{p}\times T_{n}-F_{p}\times F_{n}}{\sqrt{\left(T_{p}+F_{p}\right)\times \left(T_{p}+F_{n}\right)\times \left(T_{n}+F_{p}\right)\times \left(T_{n}+F_{n}\right)}}\times 100$$

False positive rate is given by:

(15) $$F_{pr}=\frac{F_{p}}{T_{n}+F_{p}}$$

$F_{1}$ score is given by:

(16) $$F_{1}score=\frac{T_{p}}{T_{p}+\frac{1}{2}\left(F_{p}+F_{n}\right)}\times 100$$

### 4.2. Results

A system for autonomously diagnosing various lung diseases in X-ray and CT image datasets is presented in this paper. Three experiments were investigated. Three pre-trained models were utilized to differentiate between COVID-19, pneumonia-viral, pneumonia-bacterial, TB, and normal X-ray images, namely, Resnet101, Inceptionv3, and InceptionResNetv2. The experiment was then carried out in order to enhance the results by replacing the fully-connected layer with an MCSVM classifier. Finally, the last experiment was carried out to demonstrate the effect of applying image SR on the performance of the proposed framework.

#### 4.2.1. Results for Dataset #1

Table 3 shows the evaluation metrics for three different models (Resnet101, Inceptionv3, InceptionResNetv2) with and without augmentation. The metrics evaluated are accuracy, sensitivity, specificity, precision, MCC, $F1_{score}$, and $F_{pr}$. Comparing the models without augmentation, it can be observed that InceptionResNetv2 achieves the highest performance in all metrics. Resnet101 performs the worst in terms of $F_{pr}$, with a value of 0.197. Inceptionv3 has the highest precision among the three models, but the lowest sensitivity. When data augmentation is applied, the performance of all three models improved significantly. InceptionResNetv2 continues to perform the best, achieving the highest scores in all metrics except for $F_{pr}$, which is the lowest for Resnet101. It can be observed that the $F_{pr}$ for all models improves significantly with data augmentation. This is because augmentation increases the amount of training data, which helps the models to better generalize to unseen data. Overall, the results prove that data augmentation has a significant positive impact on model performance. InceptionResNetv2 is the best-performing model in this case.

**Figure 7:**
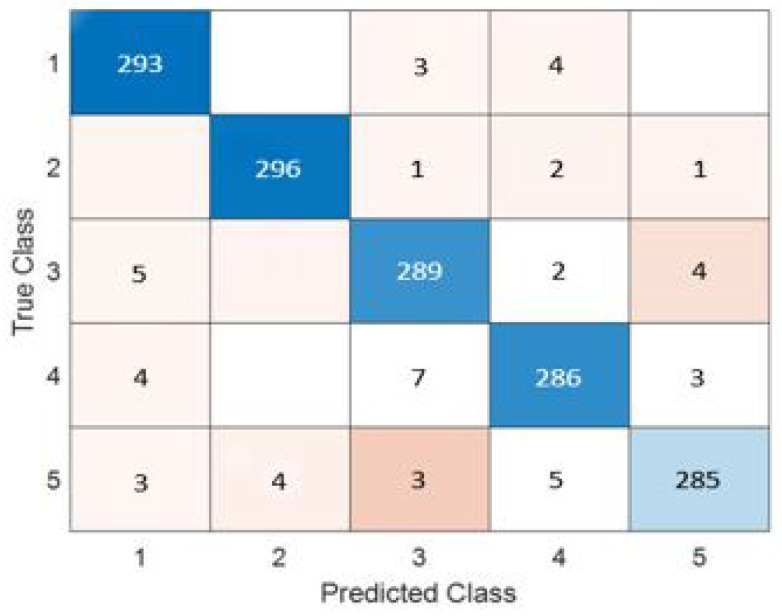
Confusion matrix for InceptionResNetv2-MCSVM-based model with image SR using dataset #1.

**Figure 8:**
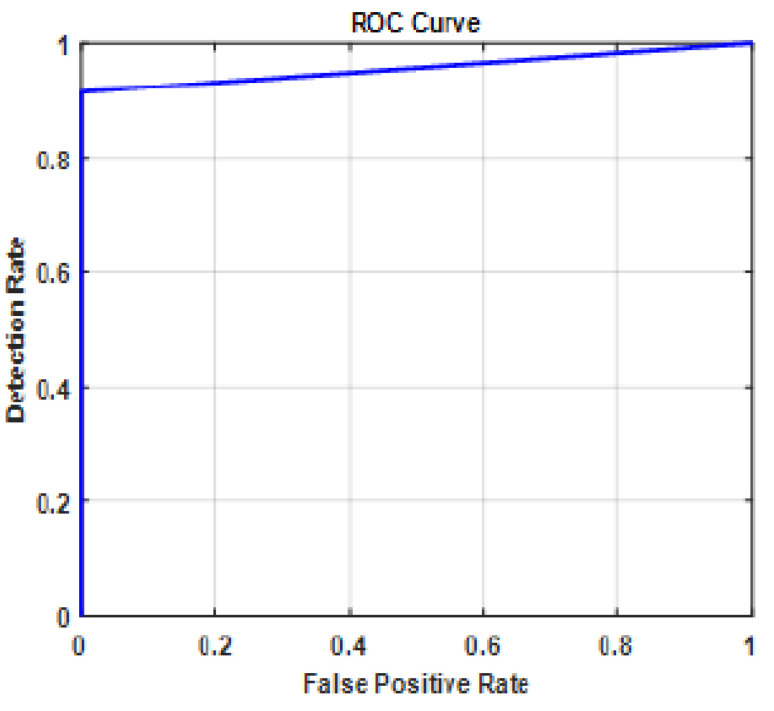
ROC curve for InceptionResNetv2-MCSVM-based model with image SR using dataset #1.

**Figure 9:**
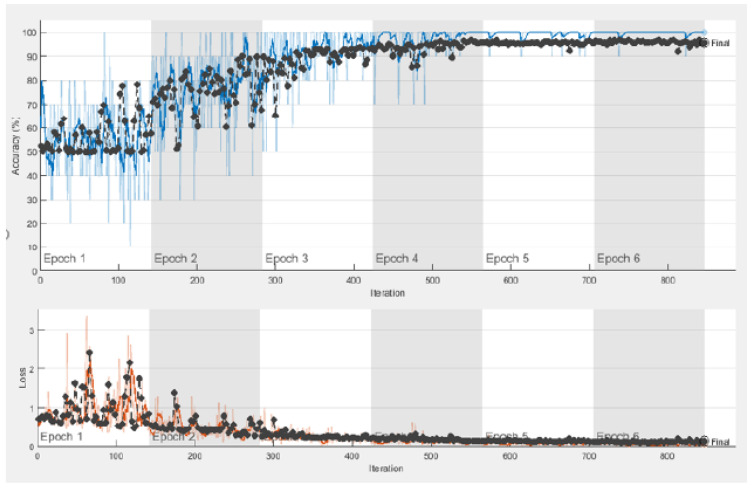
Training progress curve for InceptionResNetv2-MCSVM-based model with image SR using dataset #1.

The fully-connected layer is replaced with the MCSVM classifier to enhance the performance of the proposed framework. Table 4 clearly shows that InceptionResNetv2 continues to outperform the other pre-trained models. Furthermore, a performance improvement equivalent to that of the previous experiment is realized. In terms of accuracy, the MCSVM classifier improves the performance by 6%. The confusion matrix and ROC curve for the InceptionResNetv2-MCSVM-based model are shown in Figure 7 and Figure 8. COVID-19, normal, pneumonia-viral, pneumonia-bacterial, and TB classes are denoted by 1, 2, 3, 4, and 5, respectively. Figure 9 presents the training progress curve for the InceptionResNetv2 model. It is clear that the model learns to minimize the error between the predicted and actual labels. At the same time, the accuracy shows an upward trend as the model improves its performance on the training data.

The previous two experiments demonstrate that the performance is insufficient. The features of all classes are quite similar, particularly for COVID-19, pneumonia-viral, and pneumonia-bacterial. To address this issue, an image SR pre-processing stage is proposed. The results for the pre-trained models with image SR using dataset #1 are presented in Table 5. The reported results show that InceptionResNetv2 outperforms ResNet101 and Inceptionv3 in terms of accuracy by roughly 5% and 3%, respectively. The results for the pre-trained-MCSVM-based models with image SR are shown in Table 6. In terms of accuracy, InceptionResNetv2 still outperforms Resnet101 and Inceptionv3 by 6% and 3%, respectively.

#### 4.2.2. Results for Dataset #2

**Figure 10:**
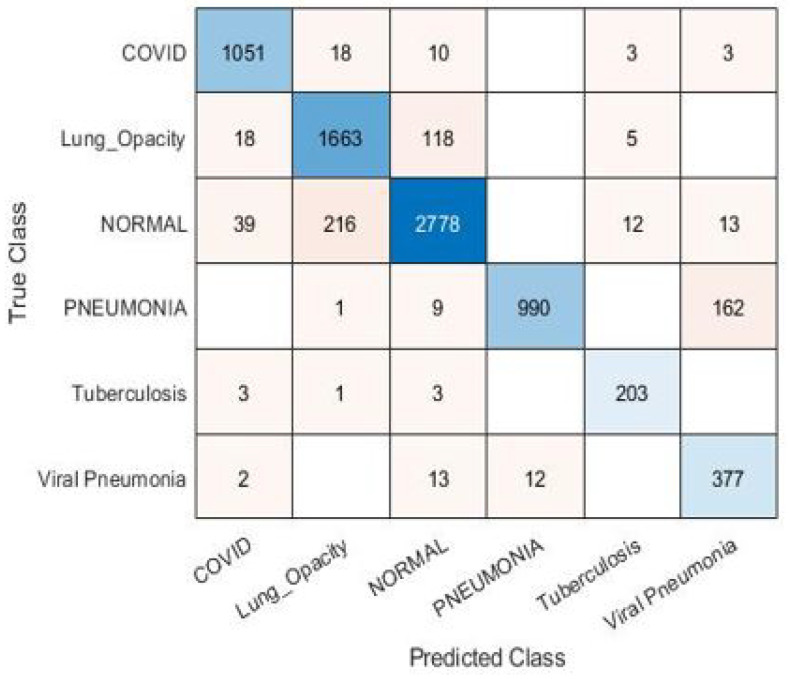
Confusion matrix for InceptionResnetv2 model with image SR using dataset #2.

**Figure 11:**
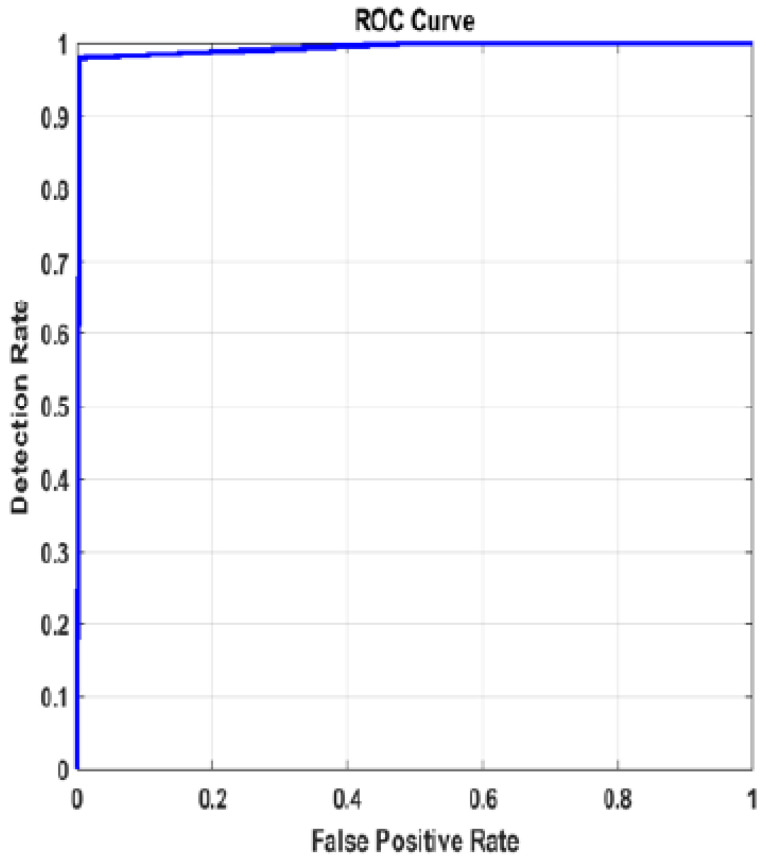
ROC curve for InceptionResnetv2 model with image SR using dataset #2.

**Figure 12:**
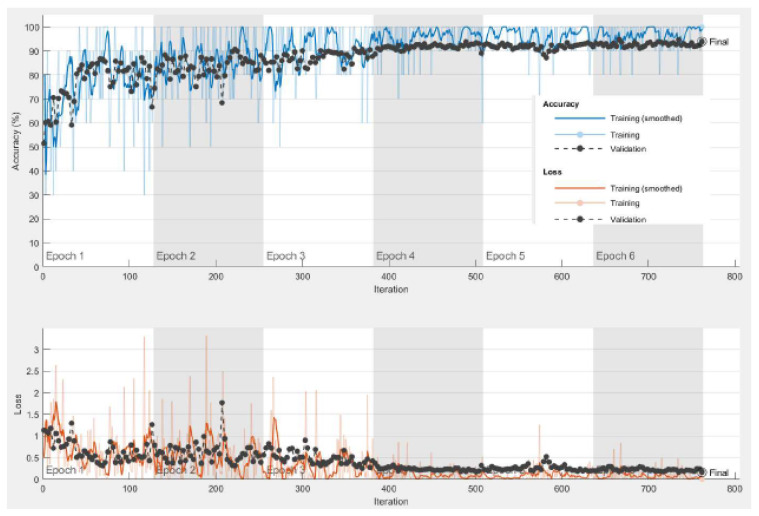
Training progress curve for InceptionResnetv2 model with image SR using dataset #2.

For the goal of generality, an X-ray dataset is composed from several publicly-available datasets for the diagnosis of lung disease. The collection contains 35,399 X-ray images from six different lung disease datasets, [61,62,63,64]. The results for the pre-trained models with image super-resolution using dataset #2 are presented in Table 7. Based on the data, it is obvious that InceptionResNetv2 outperforms ResNet101 and Inceptionv3 in terms of accuracy by roughly 1%. The confusion matrix and ROC curve for the InceptionResNetv2 model with image SR using dataset #2 are shown in Figure 10 and Figure 11. Figure 12 illustrates the progress of training for the InceptionResNetv2 model. The figure demonstrates that the model gradually reduces the difference between predicted and actual labels, leading to a decrease in error. Additionally, the accuracy of the model increases over time, indicating an improvement in its performance on the training data.

Furthermore, the experiment is performed with the MCSVM classifier rather than softmax. The results for the pre-trained-MCSVM-based models with image super-resolution using dataset #2 are presented in Table 8. The obtained results show that InceptionResNetv2 outperforms ResNet101 and Inceptionv3 in terms of accuracy by around 2%.

#### 4.2.3. Results for Dataset #3

**Figure 13:**
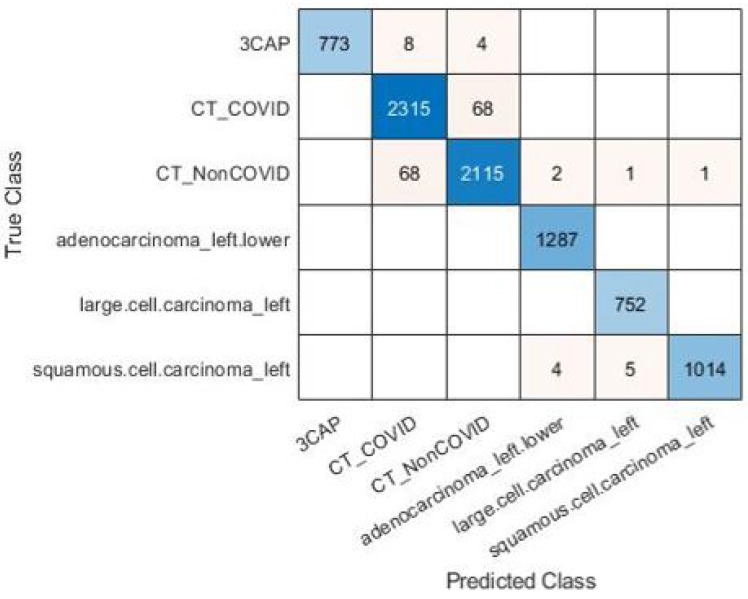
Confusion matrix for InceptionResNetv2 model with image SR using dataset #3.

**Figure 14:**
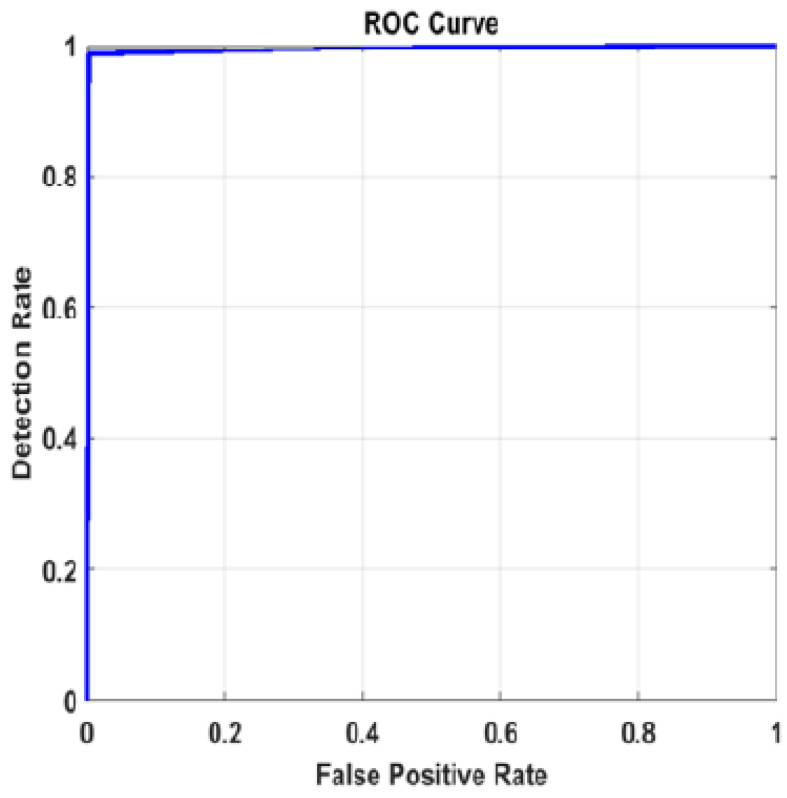
ROC curve for InceptionResNetv2 model with image SR using dataset #3.

**Figure 15:**
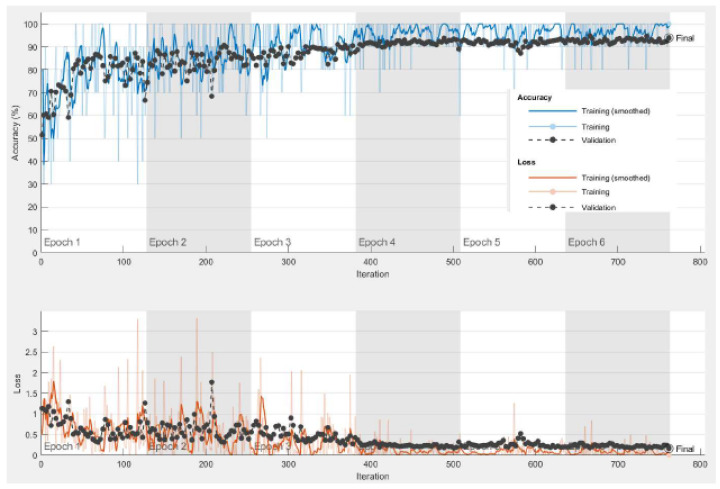
Training progress for InceptionResNetv2 model for dataset #3.

The experiment was repeated using the large CT dataset #3 to demonstrate the validity of the proposed framework. It was constructed from six publicly available datasets [64,65,66], and it has 28,058 CT scans. The results for the pre-trained models and the pre-trained-MCSVM-based models are presented in Table 9 and Table 10, respectively. The obtained results clearly indicate a decrease in the performance of the MCSVM-based models. Figure 13 and Figure 14 show the confusion matrix and ROC curve for InceptionResNetv2-MCSVM-based models using CT images. Figure 15 shows the accuracy and loss performance of the pre-trained InceptionResNetv2 model. Validation and training accuracy, as well as validation and training loss, have similar behaviour.

## 5. Discussion and Comparison with the-State-of-the-Art Methods

**Figure 16:**
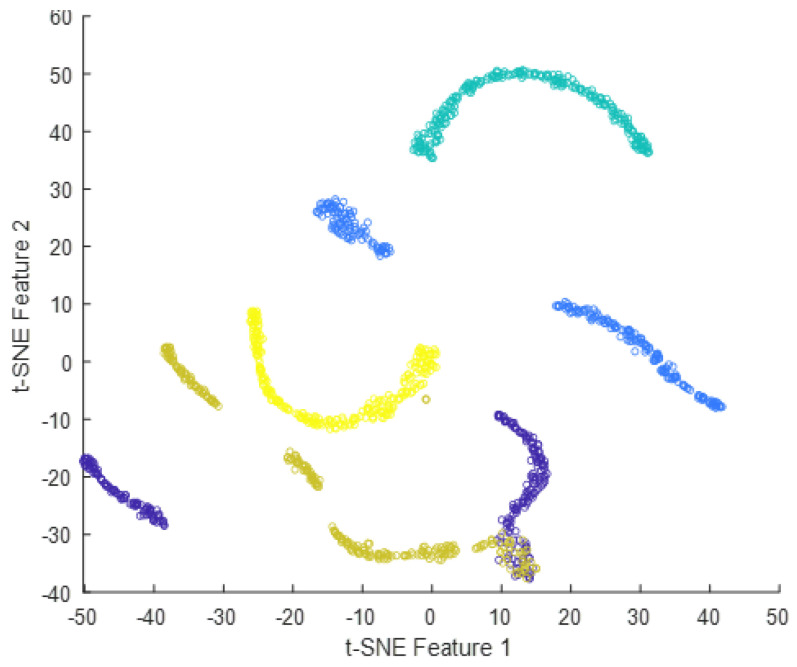
t-SNE plot of the extracted features from the fully-connected layer for dataset #1.

**Figure 17:**
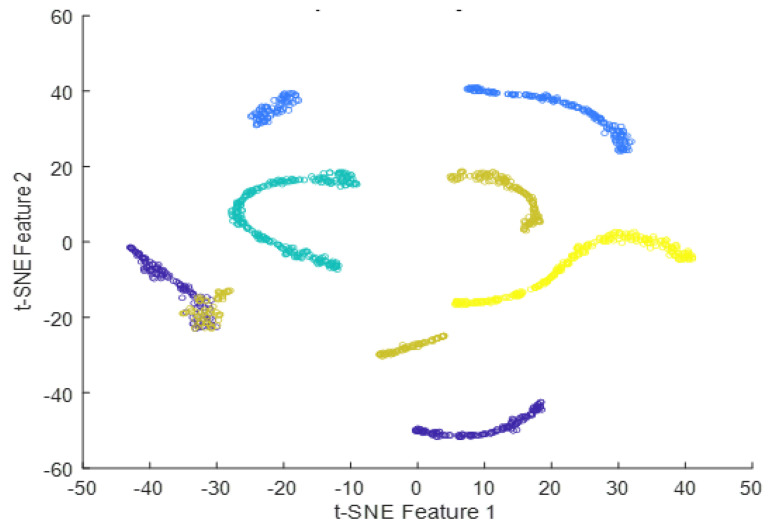
t-SNE plot of the extracted features from the fully-connected layer for dataset #2.

**Figure 18:**
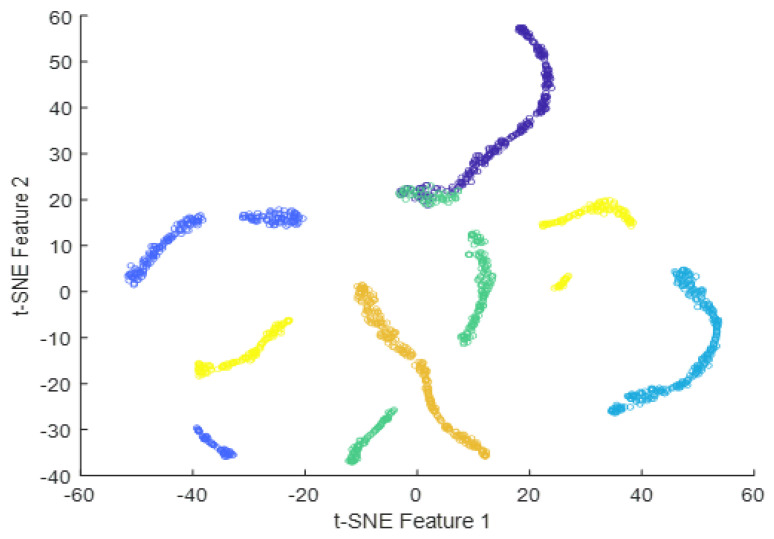
t-SNE plot of the extracted features from the fully-connected layer for dataset #3.

Deep features can be obtained from the output of any intermediate layer in a deep neural network. Each layer in a neural network learns a hierarchy of increasingly complex and abstract features from the input data. The deeper the layer, the higher the level of abstraction and complexity of the learned features is. Typically, the output of the last layer before the final fully-connected layer is used as the deep features for a given input image. The final fully-connected layer is often task-specific and may not generalize well to other tasks. The output of the last layer before the final fully-connected layer can be considered a more general feature representation that can be used for a variety of tasks, such as image classification, object detection, and image retrieval. In the case of InceptionResNetV2, the output of the global average pooling layer, which is typically the layer immediately preceding the final fully-connected layer, can be used as the deep features for the input image. This feature vector contains the most important information about the input image learned by the network and can be used for a variety of downstream tasks. In summary, the final fully-connected layer in InceptionResNetV2 takes the global feature vector obtained from the previous global average pooling layer as input, applies a linear transformation followed by an activation function, and produces the final output predictions for the given classification task. The tSNE plots of the extracted features from the fully-connected layer for dataset #1, dataset #2, and dataset #3 using InceptionResNetv2 are presented in Figure 16, Figure 17 and Figure 18. The tSNE plots reveal the relationships between different classes in the dataset. There is an overlapping cluster between different classes, and this indicates that the fully-connected layer has learned features that are shared between those classes. On the other hand, if the tSNE plot shows well-separated clusters between different classes, this indicates that the fully-connected layer has learned the features that are specific to each class.

It is clear from Table 11 that InceptionResNetv2 combined with softmax is a very strong architecture that achieves a state-of-the-art performance level on a number of image recognition tasks. This is due to its ability to capture complex patterns in the input images through the use of deep residual networks and a combination of convolutional and pooling layers. In addition, the SVM is another popular tool for image recognition tasks, particularly for its ability to handle non-linear data by mapping it to a higher-dimensional space. However, in some cases, SVM may not perform as well as deep neural networks such as InceptionResNetv2, especially when working with very large datasets or complex image recognition tasks.

**Figure 19:**
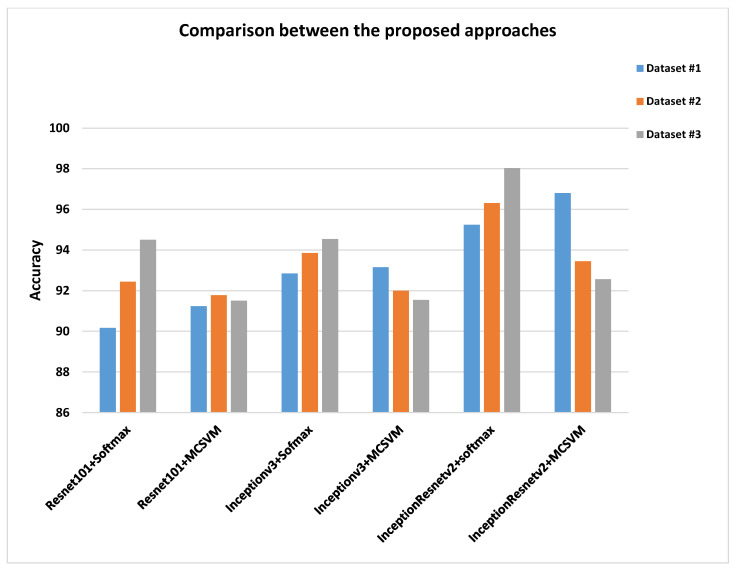
Accuracies rates for the proposed approaches.

Resnet101, Inceptionv3, and InceptionResNetv2 models were employed in simulation studies for lung disease diagnosis using three distinct datasets. As shown in Figure 19, a comparison of the obtained results with all models reveals that the InceptionResNetv2 model outperforms the Resnet101 and Inceptionv3 models. The DL-SR-based model is applied on the original images to improve the results even more. This has led to higher classification results. The use of L2-regularization yields better results than those of the softmax layer using dataset #1. Softmax outperforms MCSVM as dataset size increases for datasets #2 and #3. The InceptionResNetv2 model high performance is related to the use of the inception block, which reduces the computational cost. The residual learning improves the classification model accuracy. This leads to improved classification results.

The computation time is the final criterion for comparing the proposed framework with other ones. It is obvious from Table 12 that deep feature extraction using Inceptionv3 takes the least time. To obtain the second-best run time, deep feature extraction with the InceptionResNetv2 model is employed. However, the use of SR ideas in this study increases the run time by around 95 s, while increasing accuracy by roughly 10%. Overall, the utilization of DL model layers to extract features for feeding them to machine learning algorithms can be an effective and efficient approach, but it requires careful selection and fine-tuning of the pre-trained model to achieve the best results.

The proposed framework achieves an accuracy level of 96.80%, which is greater than the levels of traditional approaches shown in Table 13. These results ensure the efficacy of the DL-SR-based procedure in performing the required classification task using an efficient classifier.

## 6. Conclusions

In this paper, we have investigated the problem of diagnosing lung diseases. Our proposed framework depends on super-resolution techniques to enhance image details before the classification process. We considered different classes of lung diseases in our classification model. InceptionResNetv2 is used for feature extraction. It is combined with a multi-class SVM for the final classification. We conducted an extensive comparison study, which includes pre-trained models, deep learning for feature extraction combined with multi-class SVM, and our super-resolution-based model for five-class and six-class classification tasks. Our simulation results demonstrate that the combination of InceptionResNetv2 with multi-class SVM, preceded by image super-resolution, achieves the highest classification accuracy of 96.8% on X-ray images and 98.028% on the CT dataset. However, this structure has the largest computational cost, but with the best quality. We proved that fine-tuning of the SVM parameters could improve the accuracy levels further, and there is still a scope for further enhancements to reduce the computational cost. Although the proposed framework offered promising results in terms of accuracy, it is unclear how much it would improve clinical outcomes for effective lung disease treatment. In addition, the proposed framework may be limited in its ability to generalize to different imaging modalities or disease categories that are not part of the training data. In addition, the proposed framework heavily relies on the availability of large and high-quality lung disease datasets. However, obtaining such data may not always be possible, especially for rare diseases, and the lack of data can limit the accuracy and effectiveness of the proposed framework. So, further research would be needed to determine the potential clinical impact of the proposed framework. Consequently, future work could include comparing our super-resolution model with other models and validating our framework for clinical use by collecting the opinions of different specialists with mean opinion score (MOS) records before commercial use. Future research directions may comprise incorporating more complex deep learning models with more layers or other architectures to further improve the accuracy of the proposed framework, extending the proposed framework to other medical imaging modalities, exploring the potential clinical impact of the proposed framework, and investigating the ability to generalize the proposed framework to other diseases.

## Figures and Tables

**Table 1:** Datasets description.

Dataset	Lung Disease					
**Dataset #1** **X-ray images**	**COVID-19**	**TB**		**Pneumonia-bacterial**	**Pneumonia-viral**	**Normal**
	259	800		900	800	1000
**Dataset #2** **X-ray images**	**COVID-19**	**Lung opacity**	**TB**		**Pneumonia-viral**	**Normal**
	3616	6012	8624		3080	10,192
**Dataset #3** **CT images**	**COVID-19**	**Adenocarcinoma**	**Large cell carcinoma**	**Squamous cell carcinoma**	**CAP**	**Normal**
	7942	4290	2508	3410	2618	7290

**Table 2:** SVM parameters.

Parameter	Value
Penalty parameter C	1.0
Kernel	Polynomial
Degree	3.0
Gamma	Scale
Tolerance	0.001
Decision function shape	One versus rest
Number of iteration	n_classes×(n_classes−1)/2

**Table 3:** Results for pre-trained models using dataset #1.

	Models	Evaluation Metrics						
		Accuracy	Sensitivity	Speci city	Precision	MCC	F1 Score	Fpr
**Without** **Augmentation**	**Resnet101**	77.24	74.53	80.77	72.19	65.85	75.16	0.197
	**Inceptionv3**	78.52	75.12	80.97	73.43	67.37	77.98	0.158
	**InceptionResNetv2**	80.86	78.23	84.67	75.72	69.57	78.12	0.148
**With** **Augmentation**	**Resnet101**	78.25	75.43	82.37	73.29	67.15	76.86	0.094
	**Inceptionv3**	80.12	77.67	86.05	75.72	70.21	78.08	0.088
	**InceptionResNetv2**	81.86	79.58	86.57	78.78	70.56	78.84	0.084

**Table 4:** Results for pre-trained-MCSVM based models using dataset #1.

Models	Evaluation Metrics						
	Acc	Sen	Spec	Preci	Mcc	F1 Score	Fpr
Resnet101	83.21	83.03	90.37	81.89	80.15	81.02	0.074
Inceptionv3	85.34	85.34	95.11	85.12	82.21	82.36	0.0489
InceptionResNetv2	86.80	87.47	96.78	87.01	83.98	86.86	0.0322

**Table 5:** Results for pre-trained models with image SR using dataset #1.

Models	Evaluation Metrics						
	Acc	Sen	Spec	Prec	Mcc	F1 Score	Fpr
**ResNet101**	90.16	89.34	95.478	90.32	89.11	90.78	0.0314
**Inceptionv3**	92.85	91.44	96.56	92.76	90.17	92.31	0.0278
**InceptionResnetv2**	95.24	95.76	96.38	96.51	92.18	95.36	0.0157

**Table 6:** Results for pre-trained-MCSVM based models with image SR using dataset #1.

Models	Evaluation Metrics						
	Acc	Sen	Spec	Preci	Mcc	F1 Score	Fpr
**Resnet101**	91.24	91.22	97.08	91.20	88.29	91.08	0.0292
**Inceptionv3**	93.15	93.14	97.72	93.14	90.85	93.11	0.0228
**InceptionResnetv2**	96.80	97.47	98.78	97.01	93.98	96.86	0.0122

**Table 7:** Results for pre-trained models with image SR using dataset #2.

Models	Evaluation Metrics						
	Acc	Sen	Spec	Prec	Mcc	F1 Score	Fpr
**ResNet101**	92.441	92.513	98.153	89.10	88.711	90.35	0.0601
**Inceptionv3**	93.85	92.64	96.86	92.20	90.02	92.56	0.0534
**InceptionResnetv2**	96.309	96.39	99.22	96.41	96.39	95.62	0.0369

**Table 8:** Results for pre-trained-MCSVM-based models with image SR using dataset #2.

Models	Evaluation Metrics						
	Acc	Sen	Spec	Prec	Mcc	F1 Score	Fpr
**ResNet101**	91.78	92.80	97.13	90.10	89.821	91.455	0.0172
**Inceptionv3**	91.99	91.94	97.08	92.45	90.98	92.87	0.0132
**InceptionResnetv2**	93.45	92.76	98.58	92.51	92.78	90.56	0.0131

**Table 9:** Results for pre-trained models with image SR using dataset #3.

Models	Evaluation Metrics						
	Acc	Sen	Spec	Prec	Mcc	F1 Score	Fpr
**ResNet101**	94.51	90.23	98.57	91.32	90.41	92.25	0.0132
**Inceptionv3**	94.54	90.62	98.69	93.21	92.13	92.34	0.0118
**InceptionResnetv2**	98.028	98.513	99.55	98.64	98.57	98.13	0.0044

**Table 10:** Results for pre-trained MCSVM-based models with image SR using dataset #3.

Models	Evaluation Metrics						
	Acc	Sen	Spec	Prec	Mcc	F1 Score	Fpr
**ResNet101**	91.51	92.23	97.57	90.32	89.41	91.25	0.0168
**Inceptionv3**	91.54	91.62	97.69	92.21	91.13	92.87	0.0131
**InceptionResnetv2**	92.56	92.16	98.52	92.31	92.67	90.78	0.0128

**Table 11:** The best results for pre-trained models with image super-resolution using the three datasets.

Dataset	Models	Evaluation Metrics						
		Accuracy	Sensitivity	Specicity	Precision	MCC	F1 Score	Fpr
**#1**	**Resnet101 + MCSVM**	91.24	91.22	97.08	91.20	88.29	91.08	0.0292
	**Inceptionv3 + MCSVM**	93.15	93.14	97.72	93.14	90.85	93.11	0.0228
	**InceptionResNetv2 + MCSVM**	96.80	97.47	98.78	97.01	93.98	96.86	0.0122
**#2**	**Resnet101 + Softmax**	92.441	92.513	98.153	89.10	88.711	90.35	0.0601
	**Inceptionv3 + Softmax**	93.85	92.64	96.86	92.20	90.02	92.56	0.0534
	**InceptionResNetv2 + Softmax**	96.309	96.39	99.22	96.41	96.39	95.62	0.0131
**#3**	**Resnet101 + Softmax**	94.51	90.23	98.57	91.32	90.41	92.25	0.0132
	**Inceptionv3 + Softmax**	94.54	90.62	98.69	93.21	92.13	92.34	0.0118
	**InceptionResNetv2 + Softmax**	98.028	98.513	99.55	98.64	98.57	98.13	0.0044

**Table 12:** Computational time of the examined approaches using dataset #1.

Laptop Specifications	Core $I_{7}$ 10th Generation, 32 bit RAM, Nvidia RTX 2070, Gpu and Hard Tera SSD with Matlab 2020b Version
**Method**	**Computational Time (s)**
**ResNet101 Features + MCSVM**	139.9
**Inceptionv3 Features + MCSVM**	130.9
**InceptionResNetv2 Features + MCSVM**	136.7
**Resnet101 + Softmax**	221.7
**Inceptionv3 + Softmax**	199.4
**InceptionResNetv2 + Softmax**	216.2
**ResNet101 Features + MCSVM + SR**	298.2
**Inceptionv3 Features + MCSVM + SR**	221.5
**InceptionResNetv2 Features + MCSVM + SR**	230.7

**Table 13:** Comparison with the state-of-the-art methods.

Authors	Task	Technique	Accuracy (%)
**Xu et al. [35]**	**Viral Pneumonia,** **Normal, and COVID-19**	**3D DL model**	86.7
**Chandra et al. [36]**	**Normal and COVID-19**	**Automatic COVID screening** **(ACoS)**	98.06
	**COVID-19 and Pneumonia**		91.23
**Rahman et al. [49]**	**Normal and Pneumonia**	**CNN-AlexNet, ResNet18,** **DenseNet201, and SqueezeNet** **TL-based models**	98
	**Normal, Bacterial pneumonia** **and Viral pneumonia**		93.3
	**Bacterial pneumonia** **and Viral pneumonia**		95
**Ferreira et al. [50]**	**Normal and Pneumonia**	**Histogram equalization+ VGG16 CNN** **+MLP classifier**	97.4
	**Bacterial pneumonia** **and Viral pneumonia**		92.1
**Jaiswal et al. [75]**	**Normal and COVID-19**	**DenseNet201** **TL-based model**	96.23
**Proposed** **model**	**Normal, COVID-19, Viral pneumonia** **Bacterial pneumonia and TB**	**SR + Inceptioesnetv2+Softmax**	95.24
		**SR + Inceptioesnetv2+MCSVM**	96.80
	**Normal, COVID-19, Viral pneumonia** **Lung opacity, Pneumonia and TB**	**SR + Inceptioesnetv2+Softmax**	96.309
		**SR + Inceptioesnetv2+MCSVM**	93.45
	**COVID-19, Non-COVID-19, Large cell carcinoma,** **Squamous cell carcinoma and CAP**	**SR + Inceptioesnetv2+Softmax**	98.028
		**SR + Inceptioesnetv2+MCSVM**	92.56

## Data Availability

Not applicable.
